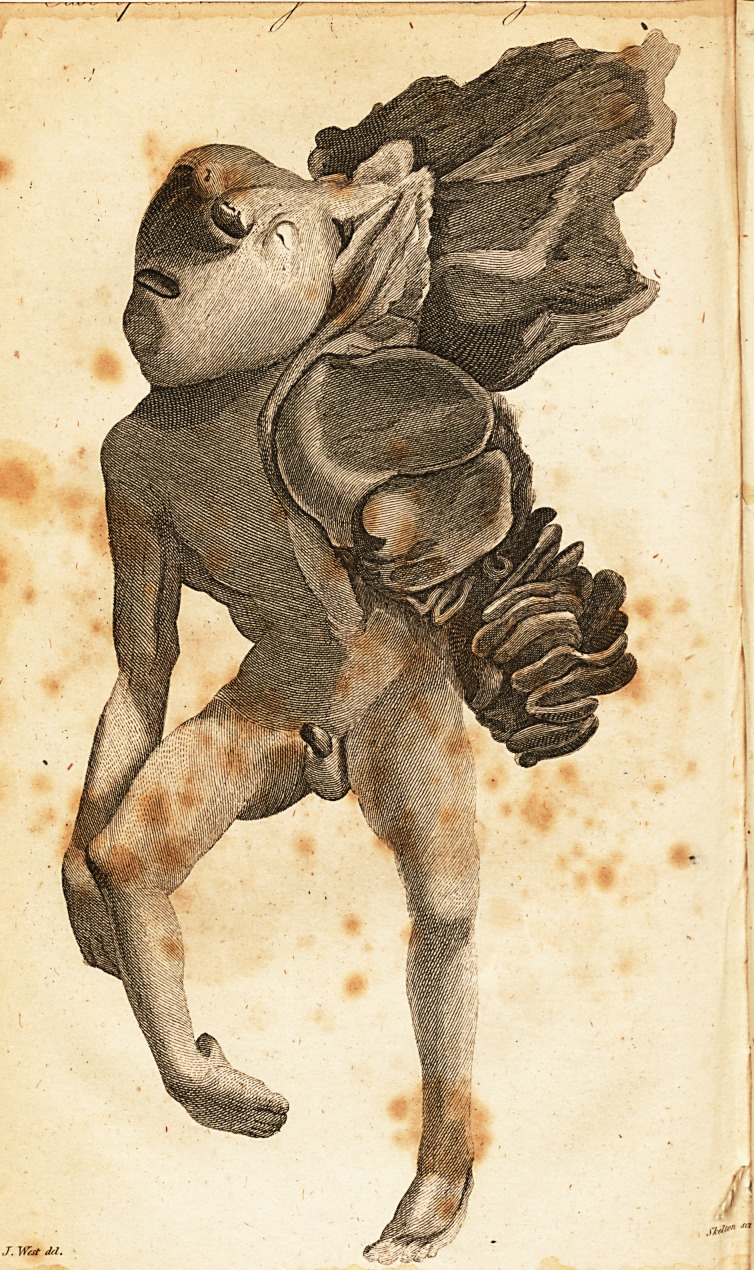# A Case of Monstrosity

**Published:** 1802-05

**Authors:** Thomas Croxall Cam

**Affiliations:** Surgeon in Bath, and late Senior Surgeon to the Hereford Infirmary


Medical and Phyfical Journal.

VOL. VII.] MAY, 1802. [NO. XXXIX.

### *A Case of Monstrosity.*

*Presented to the Editors of the Medical and Physical Journal; by* THOMAS CROXALL CAM, *Surgeon in Bath, and late Senior Surgeon to the Hereford Infirmary.*

[ With an Engraving. ]

IN April laft I was defired to vifit a Mrs. F. of this city. She had pains which appeared to be thofe of labour, but was ignorant of her fituation, from having had a difcharge of blood from the uterus, more or lefs, for fourteen weeks, which weakened her very much, but did not confine her till the day before I faw her, when the hæmorrhage was increafed by her pains.

I examined the abdomen, and difcovered a large circumfcribed tumour, much elevated, but not yielding to the touch, on the left fide, and in feel refembling a r[illegible]ained placenta. During a pain I made myfelf acquainted with the ftate of the uterus, and found the os tincæ, which I could juft reach with my finger, extremely rigid, and very little dilated. The patient had been attended the day before by an Accoucheur, who gave her aftringents with a view of checking the difcharge; but being fatisfied the veffels were not in a ftate to contract, and pains remaining, I thought it moft advifable to affift Nature in the work fhe was labouring to complete, by endeavouring to remove the rigidity of the os tincæ. Relaxing means were adminiftered, and in the courfe of the day, a male monfter was expelled by natural efforts, which I judged to be fix months old, from the placenta being two-thirds of its ufual dimenfions at the lateft period of pregnancy, but the fœtus reduced in bulk by the long continued drain.

In this curious cafe of Monftrofity the bones of the cranium were wanting.

There was not any brain, or the leaft veftige of medulla oblongata. A ragged membranous open pouch coming from the fcalp, hung down on the back.

The orbitar proceffes, and that part which forms the nafal, were perfect, and alfo the maftoid and zygomatic.

The face was regularly fhaped, but the eyes feemed to ftand on the top of the forehead from the failure of the cranium.

A portion of the membranous part of the placenta was united to the ſcalp above the left eye. The funis was not more than three inches in length. There was no clavicula, ſcapula, humerus, or any arm on the left ſide. The dorſal vertebræ were much diſtorted. There was a deficiency of the peritonæal covering of the epigaſtric region of the abdomen; the liver and ſmall inteſtines protruded from the cavity on the left ſide, and the large ones were diſtended with meconium. The right foot was inverted. The features, the extremities, and parts of generation, were large in proportion to the ſize of the monſter. The mother could not attribute this malconformation to any particular cauſe. She was greatly debilitated for a fortnight from her delivery, but ſoon after recovered her ſtrength by the aſſiſtance of the bark and a nutritious diet.

*Bath, March 3, 1802.* T. C. CAM.

---

## Figures and Tables

**Figure f1:**